# Transient Increase in AT_1_R Expression at the Myocardial Infarct Site Is Associated with Early Fibrotic Remodeling in Infarcted Rat Heart

**DOI:** 10.3390/ijms27093999

**Published:** 2026-04-29

**Authors:** Gergana O. Drumeva, Daniil R. Petrenyov, Cen Chen, Shant Der Sarkissian, François B. Tournoux, Nicolas Noiseux, Jean N. DaSilva

**Affiliations:** 1 Centre de Recherche du Centre Hospitalier de l’Université de Montréal (CRCHUM), Montréal, QC H2X 0A9, Canada; 2 Department of Pharmacology and Physiology, Université de Montréal, Montréal, QC H3C 3J7, Canada; 3 Department of Surgery, Université de Montréal, Montréal, QC H3C 3J7, Canada; 4 Department of Cardiology, Centre Hospitalier de l’Université de Montréal, Montréal, QC H2X 0C1, Canada; 5 Department of Radiology, Radiation Oncology and Nuclear Medicine, Faculty of Medicine, Université de Montréal, Montréal, QC H3C 3J7, Canada

**Keywords:** heart failure, heart function, cardiac remodeling, ischemia reperfusion, permanent ligation, fibrosis, collagen, angiotensin II type 1 receptor, echocardiography, autoradiography

## Abstract

Myocardial infarction initiates complex remodeling processes involving the renin–angiotensin system, through activation of the angiotensin II type 1 receptor (AT_1_R). This study correlated AT_1_R expression with fibrosis and cardiac function in the heart and kidneys following cardiac ischemic injury in animal models. Male Sprague-Dawley rats underwent Sham surgery, Ischemia/Reperfusion (I/R, 20-min ligation) or Permanent Ligation (PL) of the left anterior descending artery. Cardiac function was assessed by echocardiography. AT_1_R expression was measured in the heart (infarct and remote areas) and kidneys (cortex, medulla) via [^125^Iodine]Sarcosine^1^-Isoleucine^8^-Angiotensin II autoradiography. Collagen deposition was evaluated through picrosirius red staining. Left ventricular (LV) ejection fraction declined in PL models but remained stable following I/R. Post-I/R, a transient increase in cardiac AT_1_R (day-3 to week-5) correlated with an increase in collagen, whereas after PL, elevations persisted through week-12. Infarct areas consistently displayed higher AT_1_R and collagen than remote areas. Renal AT_1_R and collagen levels were unchanged across groups. In analyses with pooled animals, cardiac AT_1_R expression correlated with collagen and inversely correlated with LV Fractional Shortening (LVFS), whereas LVFS inversely correlated with collagen deposition. These findings suggest that cardiac AT_1_R levels may serve as a target of cardiac remodeling, while changes in renal AT_1_R appear less pronounced.

## 1. Introduction

Heart diseases remain one of the leading causes of mortality worldwide [1], primarily due to the detrimental effects of acute myocardial infarction (MI) [2]. Oxidative stress, apoptosis, inflammation and cardiac fibrosis are key mechanisms triggered during ischemia that persist throughout the reperfusion phase [3,4]. These progressive alterations can eventually lead to heart failure (HF), a complex clinical syndrome characterized by maladaptive remodeling, with cardiac hypertrophy, fibrosis and diastolic/systolic dysfunction [5]. Dysregulation of the renin–angiotensin system (RAS), particularly the activation of myocardial Angiotensin II type 1 Receptor (AT_1_R), plays a critical role in left ventricular (LV) remodeling and contributes to HF development [6,7]. Its activation is associated with vasoconstriction, inflammation, oxidative stress, fibrosis and hypertrophy [8]. Angiotensin II (Ang II) induces the activation of cardiac fibroblasts via AT_1_R by promoting Transforming Growth Factor-β1 (TGF-β1) production and triggering downstream signaling pathways that lead to extracellular matrix (ECM) protein deposition [9]. An increase in myocardial stiffness is a hallmark of cardiac fibrosis, resulting from excessive fibroblast activation and the accumulation of ECM components [10]. Elevated mechanical stiffness further promotes collagen deposition and fibroblast activation, reinforcing a vicious cycle that drives further fibrotic progression, ultimately leading to long-term cardiac fibrosis and impaired contractile and relaxation functions, and contributing to HF [11,12].

Despite extensive research on the role of RAS in cardiac injury, the temporal dynamics of AT_1_R expression and collagen deposition following ischemia–reperfusion injury and permanent ischemic injury remain poorly understood, particularly in relation to kidney involvement. This interplay, known as cardiorenal syndrome, is a complex and bidirectional pathological condition in which heart or kidney dysfunction triggers damage in the other organ, leading to a vicious cycle that significantly contributes to morbidity and mortality [13].

The aim of this study was to correlate temporal AT_1_R expression with fibrosis and cardiac function in the heart and kidneys following ischemic injury in two rat models of myocardial ischemia–reperfusion (20 min of coronary ligation followed by reperfusion, I/R) and permanent ligation (PL). The main outcome measure was longitudinal AT_1_R expression in the heart and kidneys. Secondary outcome measures included longitudinal cardiac and renal collagen deposition and cardiac functional parameters. A better understanding of these mechanisms could provide a rationale for targeting the RAS pathway to alleviate fibrosis and preserve organ function after ischemic events.

## 2. Results

### 2.1. Cardiac Remodeling: LV Ejection Fraction (LVEF) and LV Fractional Shortening (LVFS) Was Significantly Reduced in PL, but Not in I/R Injury in Rats

**Figure 1:**
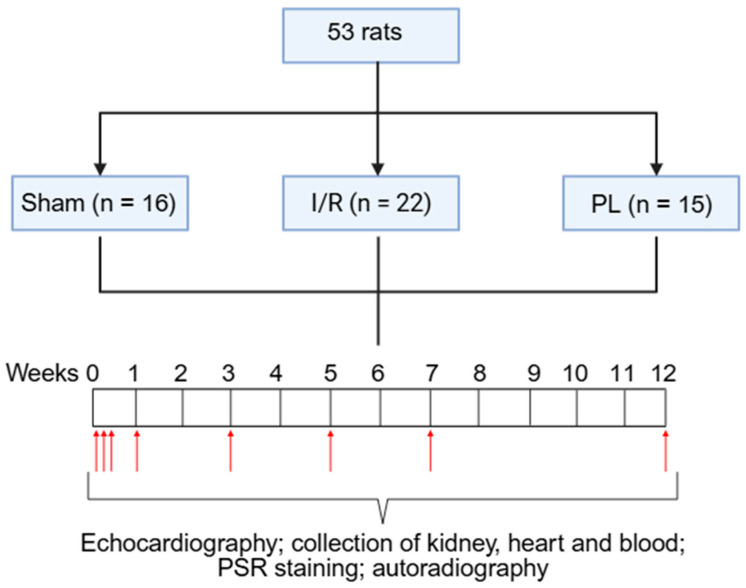
Schematic overview of the study design and timeline. Sham, Ischemia/Reperfusion (I/R; 20 min ligation) and Permanent Ligation (PL) surgeries were performed at day-0. Echocardiography was performed on a weekly basis. Animals were sacrificed at the following time points (indicated by red arrows): t = 2 h, 1 day, 3 days, 1, 3, 5, 7 and 12 weeks post-surgeries. Heart, kidney and blood samples were collected. Picrosirius red (PSR) staining and in vitro autoradiography were performed. Created in BioRender (https://BioRender.com/tzds6md; accessed on 25 February 2026).

**Figure 2:**
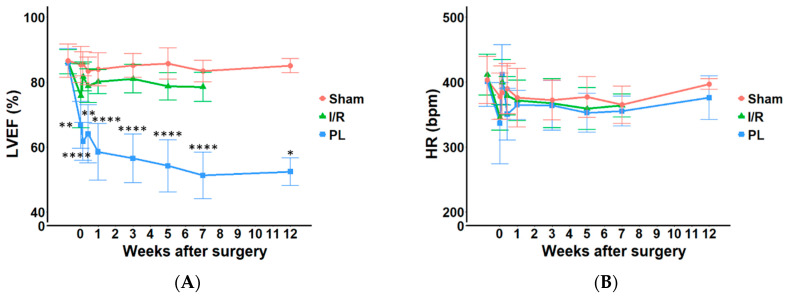
Temporal changes in cardiac function. Changes in (**A**) Left ventricular ejection fraction (LVEF, %), (**B**) Heart rate (HR) (bpm) in response to I/R and PL surgeries as compared with Sham rats. Data were analysed using one-way ANOVA followed by Dunnett’s multiple comparisons test and unpaired two-tailed *t*-test (for 12 w) (GraphPad Prism, Version 8.1.0) and plotted in R (Version 4.3.0). Data at each time point represent all available measurements from all living animals at that specific time point. Data are presented as means ± SD. *, **, **** All groups vs. Sham. * *p* ≤ 0.05; ** *p* ≤ 0.01; **** *p* ≤ 0.0001.

To evaluate the impact of PL and I/R of the left anterior descending (LAD) coronary artery on cardiac function, serial echocardiography was performed at multiple time points over a 12-week period (Figure 1 and Figure S1, Table S1). Rats subjected to PL presented sustained and significant reduction in LVEF as early as 2 h post-surgery, continuing through week-7 (Figure 2A). Specifically, LVEF declined from 85.8 ± 4.4% at baseline to 66.8 ± 7.2% (*p* < 0.01) at 2 h, 61.7 ± 5.8% (*p* < 0.0001) at day-1 and 64.0 ± 9.0% (*p* < 0.01) at day-3. This reduction persisted at later time points, with LVEF decreasing from 58.5 ± 8.8% at week-1 to 52.4 ± 4.3% at week-12.

Similarly, LVFS was profoundly reduced as early as 2 h after PL (50.4 ± 7.3% in Sham vs. 32.9 ± 5.2% in rats subjected to PL, *p* < 0.05) (Figure S2A). This reduction persisted throughout the study, with LVFS at week-12 measuring 49.8 ± 2.5% in Sham vs. 24.1 ± 2.4% in rats subjected to PL (*p* < 0.0001). In contrast, rats with I/R injury exhibited no significant differences in LVEF or LVFS compared to Sham throughout the study. Interestingly, heart rate (HR) remained unchanged in response to both permanent and transient LAD artery ligation across all groups (Figure 2B). Animals subjected to PL surgery exhibited a decrease in interventricular septum thickness in diastole (IVSd) (Figure S2B) and an increase in LV internal diameter in diastole (LVIDd) (Figure S2C) and LV internal diameter in systole (LVIDs) (Figure S2D), with no change observed in LV posterior wall thickness in diastole (LVPWd) (Figure S2E). In contrast, no significant changes were detected in any of these parameters in the I/R group (Figure S2). LV weight to body weight ratio did not differ across groups as displayed in Figure S3.

### 2.2. Increased Collagen Deposition in the Heart, but Not in the Kidneys

**Figure 3:**
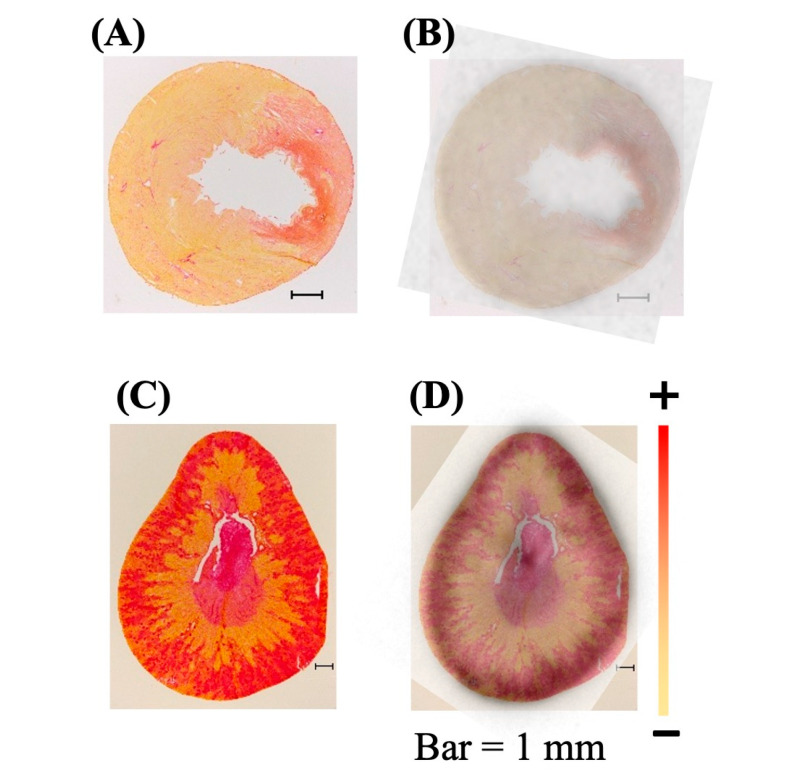

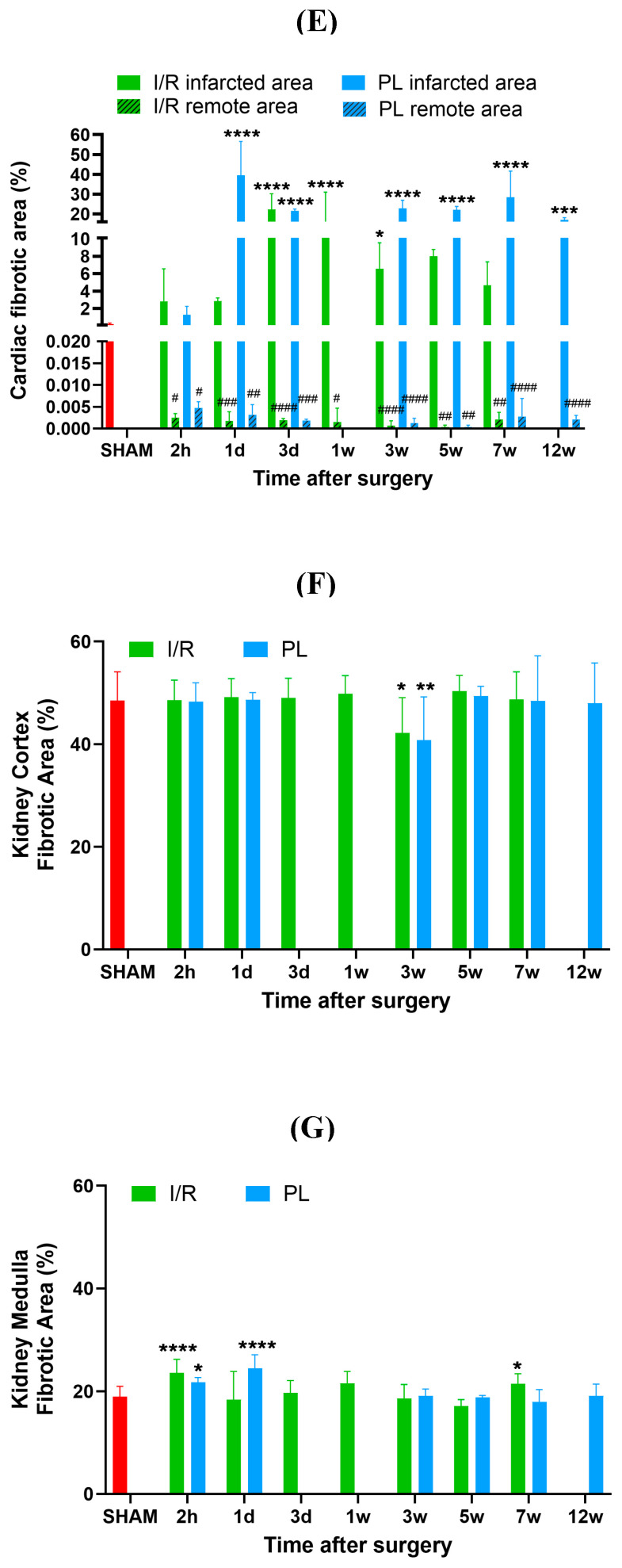
Structural remodeling in the heart and kidneys. Representative images of PSR staining in (**A**) the heart at day-1 post PL surgery and in (**C**) the kidneys at week-3 post I/R surgery. Elevation of fibrosis towards the « + ». Superimposition of the representative autoradiographic AT_1_R image and the PSR staining image in (**B**) the heart and (**D**) the kidney. Quantification of fibrosis in the heart displayed as (**E**) an expanded view providing a more detailed visualization, as well as in the kidney (**F**) cortex and (**G**) medulla. Data are presented as means ± SD of technical triplicates of all the animals per time point. Statistical significance was determined by One-Way ANOVA with Dunnett’s multiple comparisons test (*, all groups compared to Sham) and paired two-tailed *t*-test (#, infarcted vs. remote area), performed in GraphPad Prism (Version 8.1.0). *–**** All groups vs. Sham (presented in red), * *p* ≤ 0.05; ** *p* ≤ 0.01; *** *p* ≤ 0.001; **** *p* ≤ 0.0001; #–#### Infarcted vs. Remote area, # *p* ≤ 0.05; ## *p* ≤ 0.01; ### *p* ≤ 0.001; #### *p* ≤ 0.0001. h: hours, d: day, w: week.

To assess the extent of fibrosis, picrosirius red (PSR) staining was performed on heart and kidney tissues (Figure 3A and Figure 3C, respectively). In general, cardiac collagen levels were higher in infarcted areas compared to remote areas in both I/R and PL models (Figure 3E). In animals subjected to I/R surgery, cardiac collagen levels in the infarcted area increased at day-3 (22 ± 8%, *p* < 0.0001), week-1 (15 ± 16%, *p* < 0.0001) and week-3 (7 ± 3%, *p* < 0.05) post-surgery, compared to Sham (0.2 ± 0.1%). In animals subjected to PL surgery, cardiac collagen deposition was evident as early as day-1 (40 ± 17%, *p* < 0.0001) and remained elevated for the rest of the study: day-3 (22 ± 1%, *p* < 0.0001), week-3 (23 ± 4%, *p* < 0.0001), week-5 (22 ± 2%, *p* < 0.0001), week-7 (28 ± 13%, *p* < 0.0001) and week-12 (17 ± 1%, *p* < 0.001). In contrast, interstitial fibrosis in the kidney cortex (Figure 3F) and medulla (Figure 3G) displayed only minor, intermittent fluctuations with no consistent or significant trends over time. 

### 2.3. Transient AT_1_R Increase in the Heart, but Not in the Kidney

**Figure 4:**
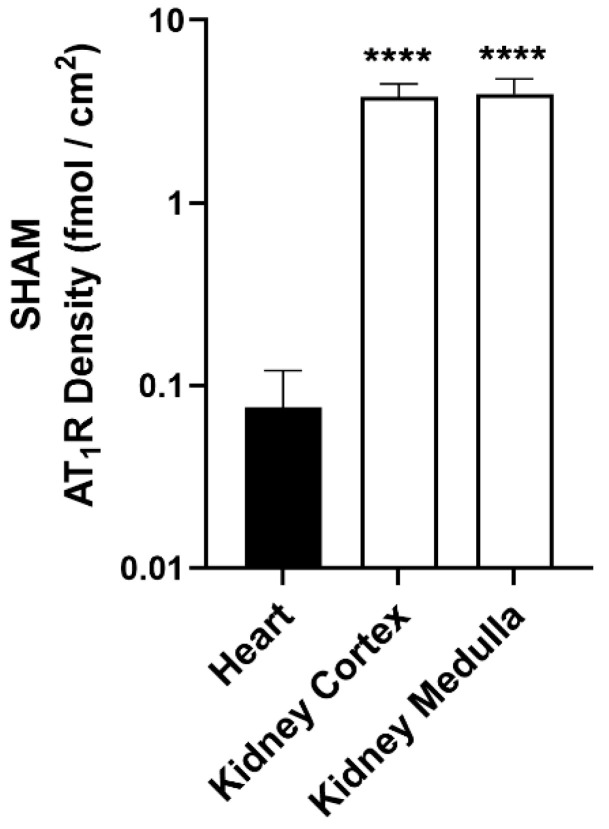
AT_1_R density in the heart, kidney cortex and kidney medulla in Sham animals. Statistical significance was determined using One-Way ANOVA followed by Dunnett’s multiple comparisons test (GraphPad Prism, Version 8.1.0). Data are presented as means ± SD. **** *p* < 0.0001, compared to the heart.

Overall, renal AT_1_R expression was significantly higher than cardiac AT_1_R expression across all groups, including Sham, I/R and PL. For example, in Sham rats, the renal cortex and medulla exhibited approximately 50-fold higher (*p* < 0.0001) AT_1_R levels compared to the heart (Figure 4). There was a colocalization between the fibrotic area and AT_1_R expression in both cardiac and renal regions as measured by the ^125^I-Sar^1^-Ile^8^-Ang II uptake (Figure 3B and Figure 3D, respectively).

**Figure 5:**
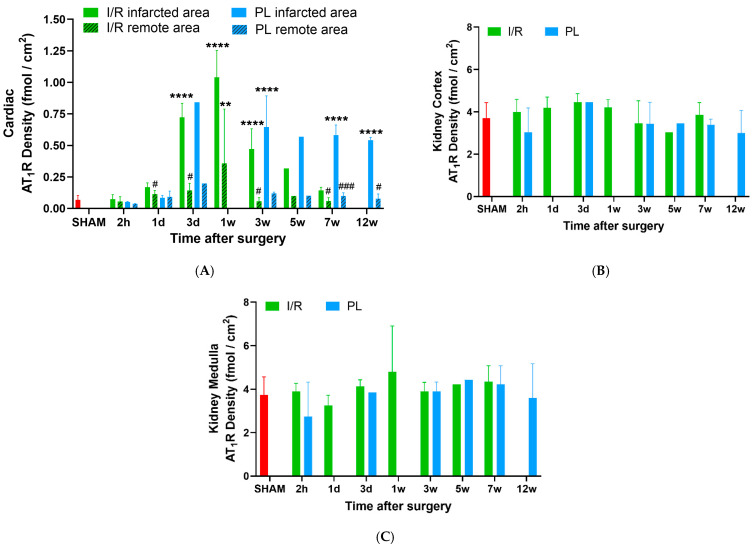
Longitudinal AT_1_R density assessed by in vitro ^125^I-Sar^1^-Ile^8^-AngII autoradiography in the (**A**) heart, (**B**) kidney cortex and (**C**) kidney medulla. Data are presented as means ± SD. Statistical significance was determined by One-Way ANOVA with Dunnett’s multiple comparisons test (*; all groups compared to Sham) and paired two-tailed *t*-test (#; infarcted vs. remote area), performed in GraphPad Prism (Version 8.1.0). **, **** All groups vs. Sham (presented in red), ** *p* ≤ 0.01; **** *p* ≤ 0.0001; #, ### Infarcted vs. Remote area, # *p* ≤ 0.05; ### *p* ≤ 0.001. h: hours, d: day, w: week.

A significant difference in AT_1_R cardiac expression was observed between infarcted and remote myocardial areas in both I/R and PL models, with remarkedly lower AT_1_R levels in the remote areas. This regional pattern remained consistent across all time points among all groups. In animals subjected to I/R, cardiac AT_1_R expression in the infarcted area increased transiently compared to Sham controls at day-3 (10-fold, *p* < 0.0001), week-1 (15-fold, *p* < 0.0001), week-3 (7-fold, *p* < 0.0001) and week-5 (5-fold, *p* < 0.05, Figure 5A). In PL animals, cardiac AT_1_R expression in the infarcted area remained elevated compared to Sham after day-3 (12-fold, *p* < 0.0001), week-3 (9-fold, *p* < 0.0001) and week-5 to week-12 (8-fold, *p* < 0.0001). In contrast, renal AT_1_R expression in both the cortex and medulla was largely unchanged across all time points in I/R and PL groups, with no significant differences compared to Sham, despite some intermittent, non-sustained fluctuations (Figure 5B and Figure 5C, respectively).

### 2.4. Cardiac AT_1_R Expression Correlated with Collagen Deposition and Inversely Correlated with Cardiac Function, While Cardiac Function Inversely Correlated with Collagen Deposition in All Pooled Animals

**Figure 6:**
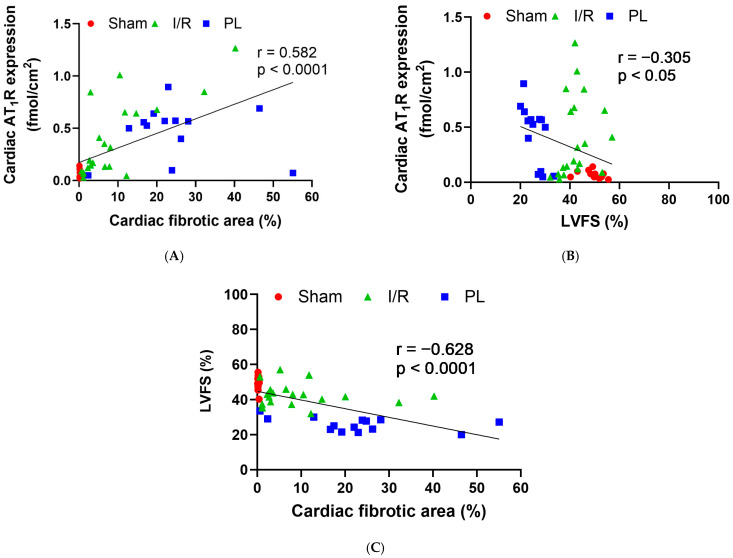
Correlations between AT_1_R expression, collagen deposition levels and LVEF in the heart of I/R and PL groups. (**A**) Correlation between cardiac AT_1_R expression and cardiac fibrotic area in all groups. (**B**) Correlation between cardiac AT_1_R expression and LVFS in all groups. (**C**) Correlation between cardiac LVFS and cardiac fibrotic area in all groups. Correlation coefficients (r) and two-tailed *p*-values were obtained using Pearson’s correlation test in GraphPad Prism (Version 8.1.0).

When analyses were performed with data pooled across all animal groups, cardiac AT_1_R expression exhibited a correlation with collagen (fibrosis) levels (r = 0.582, *p* < 0.0001; Figure 6A). Cardiac AT_1_R expression also exhibited an inverse correlation with LVFS (r = −0.305, *p* < 0.05; Figure 6B). An inverse correlation was also noted between LVFS and collagen (fibrosis) levels (r = −0.628, *p* < 0.0001; Figure 6C). As expected, inverse correlations were also observed between LVEF and cardiac AT_1_R expression (r = −0.337, *p* < 0.05; Figure S4A), as well as LVEF and collagen (fibrosis) levels (r = −0.653, *p* < 0.0001; Figure S4B).

## 3. Discussion

To the best of our knowledge, this study is the first to characterize the temporal dynamics of pathophysiological responses in cardiac function, AT_1_R expression and fibrosis following transit I/R compared to permanent LAD artery ligation. This study demonstrated that PL led to sustained reductions in cardiac function with extensive collagen deposition whereas I/R induced an early increase in collagen without long-term functional impairment. These distinct outcomes likely reflect different modulations of neurohormonal and fibrotic pathways, particularly involving the RAS, in response to varying patterns of ischemic injury. The absence of altered renal AT_1_R expression in these animal models adds key findings to the cardiorenal physiological research in myocardial ischemic injury.

Permanent coronary occlusion in PL animals caused a rapid and lasting decline in LVEF and LVFS, consistent with pathological cardiac remodeling resulting in an increased cardiac AT_1_R expression and collagen deposition in infarcted areas. This aligns with findings from both animal [14] and clinical [15] studies reporting a decline in LVEF accompanied by a sustained elevation in myocardial fibrosis. Once activated by cardiac injury, AT_1_R signaling triggers the upregulation of several downstream effectors including NADPH oxidase and TGF-β1, which leads to myofibroblast activation and ECM deposition [16,17]. Pooled analyses displayed a significant correlation between AT_1_R expression and collagen levels, indicating an association between sustained AT_1_R stimulation and persistent fibrosis, further promoting inflammatory and fibrotic cascades during the early post-infarction phase [18,19]. Alternatively, the persistence of fibrosis during the chronic post-infarction phase can occur via AT_1_R-independent mechanisms, including aldosterone-driven mineralocorticoid receptor activation and purinergic receptor signaling, as well as endothelin (ET) receptor stimulation with ET-1 [20,21,22].

In contrast to PL, I/R induced early collagen deposition and transient AT_1_R upregulation in the infarcted areas without significant reductions in LVEF or LVFS. It is important to note that the severity of myocardial injury following I/R correlates with the duration of ischemia. For instance, Michael et al. demonstrated that extending the ischemic duration to 2 h significantly increased infarct size in the I/R injury in mice to approximately 30%, closely resembling the infarct sizes observed in the PL group within the same study [23]. Similarly, a previous study has shown that prolonged ischemic durations exacerbate scar fibrosis and lead to further declines in cardiac function [24]. Across pooled animals, AT_1_R expression correlated with collagen deposition, supporting its association with ECM remodeling. This finding aligns with previous work [25] confirming that ischemia can activate the RAS signaling pathway, promoting fibroblast differentiation and collagen deposition in the early post-injury phase. Interestingly, it has previously been reported that LVEF may not exhibit significant changes in some animal models of I/R injury, particularly in the short term or under specific experiment conditions [24]. In this study, the 20-min ischemia did not lead to a decline in cardiac function despite the transient increase observed in AT_1_R. Consistent with these observations, Michael et al. [23] reported better preservation of cardiac function in animals subjected to I/R injury compared with the PL group. These findings are in agreement with this observation and highlight the protective role of reperfusion in salvaging viable myocardium [26].

Consistent with previous published studies [27], renal AT_1_R expression was significantly higher than cardiac expression under all experimental conditions yet remained unchanged in response to either PL or I/R surgeries. Abundantly expressed in renal tubular epithelial cells and vascular smooth muscle cells [28,29], renal AT_1_R is critical for regulating vasoconstriction and promoting sodium and water retention. While AT_1_R is also present in the heart, its role is more closely linked to pathological stress responses, including hypertrophy, fibrosis and inflammation. Following MI, AT_1_Rs have been reported to increase at the infarcted area in rats [30,31]. AT_1_Rs are localized not only on cardiomyocytes and cardiac endothelial cells [6,32] but also on myofibroblasts within the infarcted area [33], where they mediate the profibrotic and vasoconstrictive actions of Ang II [34]. The findings presented here are consistent with the literature, reporting a clear colocalization of AT_1_R expression with fibrotic areas in the heart, further indicating that AT_1_R expression is associated with post-infarction cardiac remodeling. Although ischemic injury can trigger systemic pathophysiological changes affecting distant organs such as the kidneys [35], consistent with cardiorenal interactions, renal fibrosis in this study remained minimal and inconsistent. This may be attributed to the fact that, despite systemic activation of the RAS, local tissue-specific regulation of AT_1_R primarily determines the extent of end-organ remodeling [36]. The heart, but not the kidney, appears particularly susceptible to injury-induced AT_1_R upregulation and its downstream effects, reflecting greater local Ang II production, higher receptor density, or the co-activation of inflammatory and oxidative signaling pathways [9,37]. Nevertheless, as the disease progresses, the kidney may also exhibit compensatory or secondary changes, leading to chronic kidney disease [38], reflecting the interactions between the heart and kidneys.

### 3.1. Implications and Future Directions

Collectively, these findings highlight the critical role of temporal regulation of AT_1_R in shaping the cardiac response to ischemic injury. The results support a model where transient AT_1_R activation contributes to reversible remodeling after I/R injury, whereas sustained AT_1_R upregulation exacerbates chronic dysfunction and fibrosis in animals subjected to PL. The AT_1_R expression pattern observed in this study aligns with prior studies demonstrating beneficial effects of AT_1_R blockade on pathological LV remodeling and fibrosis in rodents and patients [39,40,41,42]. Although mRNA does not always reflect protein expression due to post-translational regulatory mechanisms, it could be assessed in future studies to provide complementary information. Additional studies focusing on AT_1_R expression and function in fibroblasts and endothelial cells, as well as its association with local RAS and ET-1 signaling, would provide valuable mechanistic insights. In addition, AT_2_R is generally increased in post-MI remodeling and in fibrotic areas of remodeling myocardium; however, findings remain controversial in mice [43], rats [25,44] and patients [45,46]. Thus, investigating AT_2_R expression, as well as the heterodimer it can form with AT_1_R [47], would provide additional mechanistic insight into cardiac remodeling. Additional studies could focus on co-staining for AT_1_R (α-SMA, CD31, cardiac troponin (cTnT)), along with fibrosis markers (Col1a1, TGF- β1, *p*-SMAD2/3) to determine if the increase in AT_1_R expression is primarily driven by fibroblasts and mechanistically associated to ECM deposition. Future research could also involve non-invasive, in vivo real-time monitoring and longitudinal assessment of AT_1_R during cardiac injury and remodeling *via* positron emission tomography (PET) imaging. Such work would enable studying disease progression and optimize RAS-targeted therapies personalizing therapeutic interventions and early identification of patients at risk for adverse remodeling. Several radiotracers, including our recently developed ^18^F-labeled tracers [48,49], have indeed been successfully applied to detect AT_1_R altered levels in infarcted myocardium and kidneys in MI and hypertensive rat models [31,50,51,52].

### 3.2. Limitations

While the findings offer valuable insights, this study has certain limitations. It is important to note that surgical MI models (using ligations of the LAD artery) do not fully replicate the injuries observed in clinical settings. A key limitation is the 20-min occlusion of the LAD artery which may predominantly produce reversible effects, whereas longer ischemia (30–45 min) is more likely to lead to irreversible injury. Therefore, our findings may not be fully applicable to more severe ischemic injuries. Renal function, blood pressure or circulating RAS components were not evaluated in this study. Such analyses would further characterize the animals and the cardiorenal implications. AT_1_R and collagen deposition were studied on adjacent tissue sections, instead of tissues coming from the same slide. Moreover, the findings in this project cannot be extrapolated to female animal models, as sex hormones may exert cardioprotective effects and hormonal status was not controlled. This study was designed as a first step in the investigation of AT_1_R expression as the bases of future larger-scale studies aimed at identifying the time points at which AT_1_R expression reaches its peak in the heart and kidneys. Identifying these critical time frames could enable future noninvasive assessment of AT_1_R dynamics, offering valuable insights into its role in remodeling and guiding the timing of therapeutic interventions for potential responders with altered AT_1_R levels.

## 4. Materials and Methods

### 4.1. Experimental Animals

All animal experiments were approved by the Institutional Animal Protection Committee of CRCHUM (protocol number: 2I18020JDSr) and conformed to the guidelines of the Canadian Council on Animal Care. All animal experiments were conducted and reported in compliance with the *Animals in Research: Reporting* In Vivo *Experiments* (ARRIVE) guidelines [53]. Male Sprague-Dawley rats (200–250 g, *n* = 87, Charles River Laboratories, Senneville, QC, Canada) were acclimated for one week upon arrival before the start of the protocol. Animals were housed two rats per cage in a temperature-controlled facility under a 12:12 h light/dark cycle, with access to standard rodent chow and water ad libitium. No experimental treatments were administered during the study, and the cages were not rotated; therefore, potential confounders related to cage position or treatment were not controlled.

Animals were allocated to experimental groups at the time of surgery based on the surgeon’s assessment of their condition and to ensure comparable group sizes at each time point. Following surgery, group allocation was confirmed based on LVEF measurements. Therefore, no formal randomization procedure was used. The personnel performing the experiments and data collection were aware of the group identities. Analyses were also conducted with knowledge of group identities. Although the study initially aimed for three animals per time point, the final sample size ranged from one to six animals per group and per time point. Animals were included in the final analysis only if their post-operative LVEF measurements were consistent with the expected phenotype associated with the surgical procedure. Animals were excluded when their LVEF values were inconsistent with the expected phenotypes (e.g., Sham-operated animals exhibited reduced LVEF comparable to PL animals, or vice versa). Thirty-four animals were excluded from the analysis: sixteen animals were excluded because their post-operative LVEF values were inconsistent with the expected phenotype; seven animals were euthanized after reaching humane endpoints, including severe lethargy, respiratory distress, major bleeding or critical cardiac abnormalities detected by electrocardiography; an additional eleven animals were excluded due to mortality during the postoperative period. The overall mortality rate in this study was 21%, which is lower than previously reported rates in similar studies [54,55]. Therefore, *n* = 53 rats were used in the final analyses (Sham (*n* = 16), I/R (*n* = 22) or PL (*n* = 15).

### 4.2. Study Design

Figure 1 illustrates the timeline of the experimental procedure. Echocardiography was performed on a weekly basis to monitor cardiac function. Animals were sacrificed at the following time points: t = 2 h, 1 day, 3 days, 1, 3, 5, 7 and 12 weeks post-surgery (*n* = 1–6 animals per groups) or earlier, if they showed signs of significant distress, including >20% body mass loss, dehydration, respiratory distress, group isolation or severe HF assessed by echocardiography. Throughout the study, all animals were closely monitored for vital signs, hydration status and signs of discomfort. Heart, kidney and blood samples were collected at the time of sacrifice. PSR staining and in vitro autoradiography were performed at each designated time point, with analyses conducted in triplicate for each animal.

### 4.3. Myocardial Infarction and Ischemia/Reperfusion Animal Models

Animal models were established as previously described [56] with representative images of the procedure illustrated in Figure S5A,B. Briefly, at least one hour before the surgery, all animals received saline (5 mL/kg s.c., 37 °C), carprofen (5 mg/kg s.c.) and buprenorphine (0.05 mg/kg s.c.). Animals were placed on a heating pad and were under general anesthesia with 4% isoflurane (Abbott, Abbott Park, IL, USA) and maintained under anesthesia with 2% isoflurane, combined with 1 L/min of oxygen with a pressure which should not exceed 20 mmHg. MI was induced by PL of the LAD coronary artery. Sham-operated animals underwent the same surgical procedure without ligation. For the I/R model, a similar surgical intervention was carried out, but after 20 min of ischemia, reperfusion was achieved by loosening the suture knot and removing the polypropylene tube PE-50 (24 g × 34 po Protect IV Plus (3063 Jelcol Catheter; Cardinal Health, Dublin, OH, USA) placed beneath the LAD. Electrocardiogram (ECG) ST-segment elevation, changes in cardiac contractility and visual observation of the myocardial blanching were used to confirm LAD ligation. To ensure a good recovery, animals were provided with a standard rodent chow moistened with the nutritional supplement Ensure. All animals received carprofen (s.c. 5 mg/kg/day) and saline (s.c. 5 mL/kg) for 3 days post-surgery.

### 4.4. Transthoracic Echocardiogram

All animals underwent a serial transthoracic echocardiogram (TTE) prior to surgery and weekly thereafter throughout the study to assess cardiac functional parameters. Echocardiographic assessments were performed under 2% isoflurane anesthesia using a Vivid 9 echocardiography machine and a 13-MHz probe (General Electric, Boston, MA, USA). Before TTE, animals were shaved and placed on a heating pad to maintain body temperature. Measurements were obtained from short-axis views using M-mode to evaluate the HR, LVEF, LVFS, IVSd, LVIDd, LVIDs and LVPWd. For each parameter, three to five representative cardiac cycles were analysed and averaged. Body weight was also recorded at the time of each TTE session.

### 4.5. Euthanasia and Necropsy

The rats were weighed and anesthetized with 3% isoflurane at 0.5 L/min O_2_ after confirming the absence of a reflex response to pinching their hind legs. At the specified time points, one final TTE was performed before sacrifice. 0.1 mL of saturated KCl was injected into the LV to arrest the heart in diastole. Euthanasia was carried out by making an incision along the abdominal plane at the white line and performing complete exsanguination through the inferior vena cava using a 25G needle, rinsed with 2% EDTA, followed by the induction of a pneumothorax. Plasma was collected by centrifuging the blood at 1000× *g* for 5 min at 4 °C before storing it at −80 °C. The hearts and kidneys were removed and stripped of fat. They were rinsed in PBS, weighed and immersed in O.C.T™ Compound (Sakura Finetek USA Inc., Torrance, CA, USA). These tissues were then frozen on dry ice and stored at −80 °C.

### 4.6. Picrosirius Red Staining

Heart tissue sections (20 µm thick) were prepared at −20 °C using a Reichert-Jung/Leica Frigocut 2800 cryostat (Leica Biosystems, Nussloch, Gremany), thaw-mounted on glass slides (Thermo Fischer Scientific, Waltham, MA, USA) and stored at −80 °C. PSR staining was carried out according to the manufacturer’s protocol, with minor modifications [57]. On the day of the experiment, slides were left at RT for a few minutes. Heart and kidney slides were rehydrated through a graded ethanol series (100, 80, 40% ethanol, 2 min each), followed by a 10-s rinse in deionized water. Slides were then fixed in 10% formalin (Thermo Fischer Scientific, Waltham, MA, USA) for 30 min and washed twice with deionized water (5 min each). Sections were incubated with PSR (Thermo Fischer Scientific, Waltham, MA, USA) for 2 h, then they were rinsed twice with acidified water (0.5% acetic acid; Sigma–Aldrich, St. Louis, MO, USA). After two brief washes in deionized water (2 min each), slides were dehydrated with 95% ethanol and 100% ethanol (two changes each, 2 min per change), cleared in xylene (two changes, 2 min each; Thermo Fischer Scientific, Waltham, MA, USA) and mounted in a resinous medium VectaMount (Vector Laboratories Inc., Burlingame, CA, USA). Stained samples were imaged at × 0.63 magnification for heart or ×0.8 magnification for kidneys using a ZEISS Stemi 508 bright field microscope (ZEISS, Oberkochen, Germany) with an integrated Axiocam 105 camera (ZEISS, Oberkochen, Germany). Fibrotic areas (red-stained regions) were quantified using ImageJ (1.54f software, NIH, Bethesda, MD, USA). PSR analysis was performed by adjusting color threshold with the following parameters: Thresholding method: Default; Threshold color: Red; color space: Lab; with the choice of a dark background. Color deconvolution was then applied to digitally separate and transform the red, green, and blue (RGB) channels of color images leading to ‘stain channels’. A 0–253 threshold was applied to the color deconvolution images to select infarcted/fibrotic area, and 2–255 was applied to select fibrotic and non-fibrotic area. Fibrosis was calculated [58,59] as a percentage of total tissue area using the following formulas: % fibrosis (scar formation) = fibrotic area/(fibrotic area + non-fibrotic area of LV). For renal interstitial fibrosis: % fibrosis = fibrotic area in the cortex or medulla/whole kidney.

### 4.7. In Vitro Autoradiography

In vitro [^125^Iodine]Sarcosine^1^-Isoleucine^8^-Angiotensin II ([^125^I]Sar^1^-Ile^8^-Ang II) autoradiography was performed as previously described [52,57]. Briefly, on the day of the experiment, slides were incubated at RT for 15 min in assay buffer (150 mM NaCl (Thermo Fischer Scientific, Waltham, MA, USA), 50 mM Na_2_HPO_4_ dibasic (VWR, Radnor, PA, USA), 1 mM EDTA (Wisent Inc., Saint-Jean-Baptiste, QC, Canada), 0.1 mM bacitracin (Thermo Fischer Scientific, Waltham, MA, USA) and 0.1% BSA (pH 7.4, VWR, Radnor, PA, USA). Slides were then incubated with 0.25 nM of ^125^I-Sar^1^-Ile^8^-Ang II (Perkin Elmer, Shelton, CT, USA) for 90 min at RT along with AT_2_R antagonist PD 123, 319 (10 µM, Alomone Labs, Jerusalem, Israel) to obtain total non-AT_2_R binding (TB), or with unlabeled Ang II (10 µM, Alomone Labs, Jerusalem, Israel) to determine non-specific binding (NSB). After incubation, slides were washed at 4 °C: twice with deionized water, four times with assay (2 min each) and again twice with deionized water. Slides were then air-dried. Heart and kidney sections were exposed to a phosphor imaging screen (Amersham Bioscience; Cytiva Life Sciences, Marlborough, MA, USA). Even though exposure times differed between the heart (5 days) and the kidneys (2 days) due to differences in signal intensity and in AT_1_R expression between organs, quantitative comparability was ensured with co-exposed calibration standards. They were included on each phosphor screen and were used to generate a calibration curve, allowing for conversion from gray levels to absolute values of radioactive concentration. This allows for reliable comparison between tissues despite the different exposure times. Calibration curves were prepared using 1.5-fold serial dilution of ^125^I-Sar^1^-Ile^8^-Ang II, ranging from 0.007 to 0.25 nM for the heart sections, and 1.7-fold serial dilution of ^125^I-Sar^1^-Ile^8^-Ang II, ranging from 0.004 to 0.5 nM for the kidney sections. The screens were scanned using an Amersham Typhoon biomolecular imager at 50 µm resolution (Cytiva Life Sciences, Marlborough, MA, USA) and images were analyzed using Image J 1.52a software (National Institutes of Health (NIH), USA). Regions of interest (ROIs; infarcted area, remote area, kidney cortex, kidney medulla) were manually outlined based on signal intensity, and ^125^I-Sar^1^-Ile^8^-Ang II uptake was quantified in fmol/cm^2^. Specific binding (SB) was calculated as: SB = TB − NSB.

### 4.8. Statistical Analysis

Data were expressed as means ± standard deviation (SD). Statistical analyses were performed using GraphPad Prism Version 8.1.0. For TTE analyses, data at each time point represent all available measurements from all living animals regardless of their sacrificing dates. Depending on the comparison between groups, either two-tailed unpaired *t*-test, two-tailed paired *t*-test or one-way ANOVA followed by Dunnett’s post-hoc test was used for multiple comparisons. Pearson’s correlation test was applied for correlation analyses. Significant difference was defined as *p* < 0.05. Figures were generated using either GraphPad Prism (version 8.1.0) or R (version 4.3.0). Statistical significance is indicated as follows: *–**** All groups vs. Sham, * *p* < 0.05; ** *p* < 0.01; *** *p* < 0.001; **** *p* < 0.0001; #–#### Infarcted vs. Remote area, # *p* ≤ 0.05; ## *p* ≤ 0.01; ### *p* ≤ 0.001; #### *p* ≤ 0.0001.

## 5. Conclusions

This study provides important insights into the temporal changes in cardiac AT_1_R expression in ischemic animal models, highlighting its potential as an early diagnostic target for HF. In animals subjected to I/R injury, there was a transient increase in cardiac AT_1_R levels and collagen deposition but no change in LVEF. In PL models, reduced LVEF was associated with increased cardiac AT_1_R levels and fibrosis. Predominant localization of cardiac AT_1_R expression to infarcted, fibrotic areas was observed in both I/R and PL models. In analyses with pooled animals, cardiac AT_1_R levels reflected fibrotic remodeling and reduced LV function, whereas renal AT_1_R and collagen expression remained unchanged. In the same analyses, reduced cardiac function was associated with increased collagen deposition. Notably, the changes were predominantly observed in animals subjected to PL surgery and were not evident in Sham or I/R groups. These findings support the potential of targeted molecular imaging of myocardial, and not renal, AT_1_R expression to provide valuable insights into regional RAS activation in HF.

## Data Availability

The original contributions presented in this study are included in the article/Supplementary Materials. Further inquiries can be directed to the corresponding author.

## Supplementary Materials

The following supporting information can be downloaded at: https://www.mdpi.com/article/10.3390/ijms27093999/s1.

## Abbreviations

Ang II	Angiotensin II
ARBs	Angiotensin receptor blockers
AT_1_R	Angiotensin II type 1 receptor
ECM	Extracellular matrix
HF	Heart failure
IVSd	Interventricular septum thickness in diastole
I/R	Ischemia/Reperfusion
LAD	Left anterior descending
LV	Left ventricular
LVEF	Left ventricular ejection fraction
LVFS	Left ventricular fractional shortening
LVIDd	Left ventricular internal diameter in diastole
LVIDs	Left ventricular internal diameter in systole
LVPWd	Left ventricular posterior wall thickness in diastole
MI	Myocardial infarction
NADPH	Nicotinamide adenine dinucleotide phosphate
PET	Positron emission tomography
PL	Permanent ligation
PSR	Picrosirius red
RAS	Renin-angiotensin-system
TGF-β1	Transforming growth factor-β1
TTE	Transthoracic echocardiogram
