# Supplementary material for: Transient Increase in AT1R Expression at the Myocardial Infarct Site Is Associated with Early Fibrotic Remodeling in Infarcted Rat Heart
Source: Int J Mol Sci. 2026 Apr 29;27(9):3999. doi: 10.3390/ijms27093999 (PMC13164082; doi:10.3390/ijms27093999)
Supplement: Supplementary file 1 [file ijms-27-03999-s001.zip › ijms-4200321-supplementary.pdf]

Supplementary Matterials

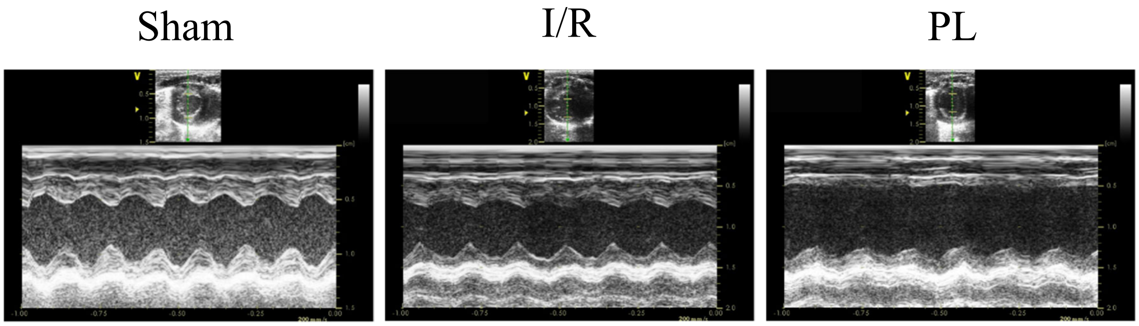

Figure S1. Temporal changes of cardiac function. Representative left ventricular echocardiographic images (short axis, M-mode) in Sham, Ischemia/Reperfusion (I/R; 20 minutes ligation) and Permanent Ligation (PL) animal models at week-3.

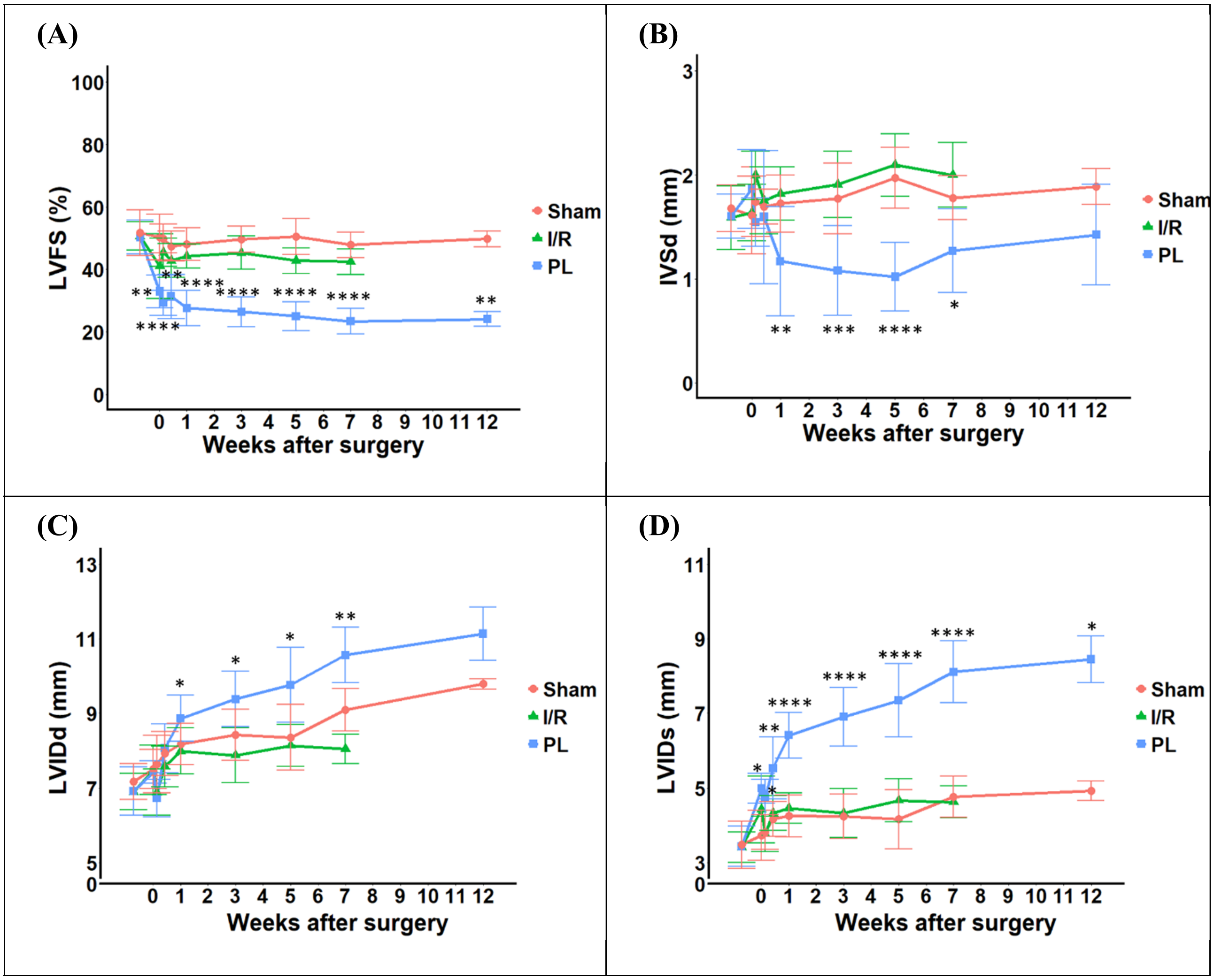

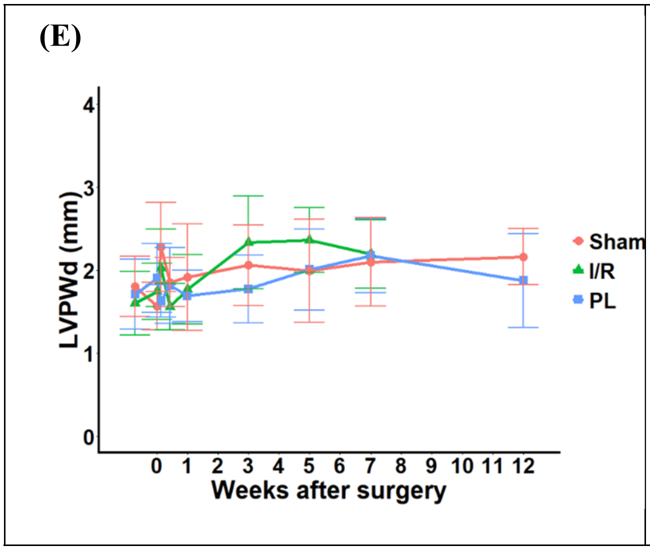

Figure S2. Changes of echocardiographic parameters following I/R and PL surgeries, as compared to Sham rats. (A) LV fractional shortening (LVFS, %) **(B)** Interventricular septum thickness in diastole (IVSd, mm). **(C)** LV internal diameter in diastole (LVIDd, mm). **(D)** LV internal diameter in systole (LVIDs, mm). **(E)** LV posterior wall thickness in diastole (LVPWd, mm). Figures were plotted in R.4.3.0 and statistical significance was determined by One-Way ANOVA with Dunnett's multiple comparisons test and unpaired two-tailed T-test (for 12w) using GraphPad Prism (Version 8.1.0). Data at each time point represent all available measurements from all living animals at that specific time point. Data are presented as means ± SD. *-**** All groups vs Sham. * $p \leq 0.05$; ** $p \leq 0.01$; *** $p \leq 0.001$; **** $p \leq 0.0001$.

Time after surgery	Baseline	2h	1d	3d	1w	3w	5w	7w	12w
	**SHAM**								
**LVEF, %**	86.6 ± 5.1	85.4 ± 5.6	85.6 ± 3.7	83.4 ± 4.3	83.9 ± 5.1	85.1 ± 3.7	85.7 ± 4.8	83.3 ± 3.3	85.0 ± 2.1
**HR, bpm**	403.0 ± 36.3	377.7 ± 35.9	383.5 ± 40.8	389.6 ± 38.9	375.6 ± 45.0	372 ± 30.6	376.9 ± 31.4	364.7 ± 28.8	396.4 ± 8.2
**LVFS, %**	51.8 ± 7.4	50.4 ±7.3	49.9 ± 4.6	47.4 ± 4.8	48.0 ±5.2	49.6 ± 4.2	50.6 ± 5.8	47.9 ± 4.1	49.8 ± 2.5
**IVSd, mm**	1.7 ± 0.2	1.6 ± 0.4	1.7 ± 0.3	1.7 ± 0.2	1.7 ± 0.3	1.8 ± 0.3	2.0 ± 0.3	1.8 ± 0.2	1.9 ± 0.2
**LVIDd, mm**	7.2 ± 0.5	7.5 ± 0.5	7.6 ± 0.8	7.9 ± 0.6	8.2 ± 0.6	8.4 ± 0.7	8.4 ± 0.9	9.1 ± 0.6	9.8 ± 0.1
**LVIDs, mm**	3.5 ± 0.6	3.7 ± 0.7	3.8 ± 0.5	4.2 ± 0.5	4.2 ± 0.6	4.2 ± 0.6	4.2 ± 0.8	4.8 ± 0.6	4.9 ± 0.3
**LVPWd, mm**	1.8 ± 0.4	1.6 ± 0.3	2.3 ± 0.5	1.9 ± 0.3	1.9 ± 0.6	2.1 ± 0.5	2.0 ± 0.6	2.1 ± 0.5	2.2 ± 0.3
	**I/R**								
**LVEF, %**	86.2 ± 3.7	75.7 ± 9.8	81.6 ± 4.4	78.8 ± 5.2	80.1 ± 3.7	81.0 ± 4.4	78.6 ± 4.2	78.5 ± 4.5	
**HR, bpm**	411.2 ± 31.4	345.5 ± 19.9	399.3 ± 35.0	378.6 ± 29.5	371.5 ± 31.3	367.1 ± 37.9	358.7 ± 32.2	363.6 ± 17.5	
**LVFS, %**	50.7 ± 4.5	41.0 ± 10.4	45.5 ± 4.5	42.9 ± 5.3	44.3 ± 3.8	45.3 ± 5.3	42.8 ± 4.1	42.5 ± 4.1	
**IVSd, mm**	1.6 ± 0.3	1.6 ± 0.3	2.0 ± 0.2	1.8 ± 0.3	1.8 ± 0.3	1.9 ± 0.3	2.1 ± 0.3	2.0 ± 0.3	
**LVIDd, mm**	6.9 ± 0.5	7.5 ± 0.7	6.9 ± 0.6	7.6 ± 0.5	8.0 ± 0.6	7.9 ± 0.7	8.1 ± 0.6	8.0 ± 0.4	
**LVIDs, mm**	3.4 ± 0.4	4.4 ± 0.9	3.8 ± 0.5	4.3 ± 0.5	4.5 ± 0.4	4.3 ± 0.7	4.7 ± 0.6	4.6 ± 0.4	
**LVPWd, mm**	1.6 ± 0.4	1.7 ± 0.3	2.0 ± 0.5	1.6 ± 0.3	1.8 ± 0.4	2.3 ± 0.6	2.4 ± 0.4	2.2 ± 0.4	
	**PL**								
**LVEF, %**	85.8 ± 4.4	66.8 ± 7.2**	61.7 ± 5.8****	64.0 ± 9.0**	58.5 ± 8.8****	56.5 ± 7.5****	54.2 ± 8.0****	51.2 ± 7.1****	52.4 ± 4.3*
**HR, bpm**	400.7 ± 38.7	336.4 ± 62.6	411.0 ± 46.1	350.2 ± 40.1	364.6 ± 22.4	363.8 ± 38.4	352.5 ± 30.2	354.9 ± 22.9	375.6 ± 33.5
**LVFS, %**	50.3 ± 5.4	32.9 ± 5.2**	29.3 ± 4.0****	31.3 ± 7.1**	27.6 ± 5.7****	26.4 ± 4.8****	25.0 ± 4.6****	23.4 ± 4.0****	24.1 ± 2.4**
**IVSd, mm**	1.6 ± 0.2	1.9 ± 0.4	1.5 ± 0.2	1.6 ± 0.6	1.2 ± 0.5**	1.1 ± 0.4***	1.0 ± 0.3****	1.3 ± 0.4*	1.4 ± 0.5
**LVIDd, mm**	6.9 ± 0.6	7.4 ± 0.3	6.7 ± 0.5	8.1 ± 0.7	8.9 ± 0.6*	9.4 ± 0.7*	9.8 ± 1.0*	10.6 ± 0.7**	11.1 ± 0.7
**LVIDs, mm**	3.4 ± 0.5	5.0 ± 0.4*	4.8 ± 0.5*	5.5 ± 0.8**	6.4 ± 0.6****	6.9 ± 0.8****	7.3 ± 1.0****	8.1 ± 0.8****	8.4 ± 0.6*
**LVPWd, mm**	1.7 ± 0.4	1.9 ± 0.4	1.6 ± 0.2	1.8 ± 0.5	1.7 ± 0.3	1.8 ± 0.4	2.0 ± 0.5	2.2 ± 0.4	1.9 ± 0.6

Table S1: Temporal echocardiographic parameters

LVEF: left ventricle ejection fraction, HR: heart rate, LVFS: left ventricle fractional shortening, IVSd: intraventricular septum thickness in diastole, LVIDd: left ventricular internal diameter in diastole, LVIDs: left ventricular internal diameter in systole, LVPWd: left ventricular posterior wall thickness in diastole. Data are

presented as means ± SD. One-Way ANOVA with Dunnett's multiple comparisons test and unpaired two-tailed T-test (for 12w) using GraphPad Prism (Version 8.1.0). Data at each time point represent all available measurements from all living animals at that specific time point. Data are presented as means ± SD. *-**** All groups vs Sham. * $p \leq 0.05$; ** $p \leq 0.01$; *** $p \leq 0.001$; **** $p \leq 0.0001$. h: hours, d: day, w: week.

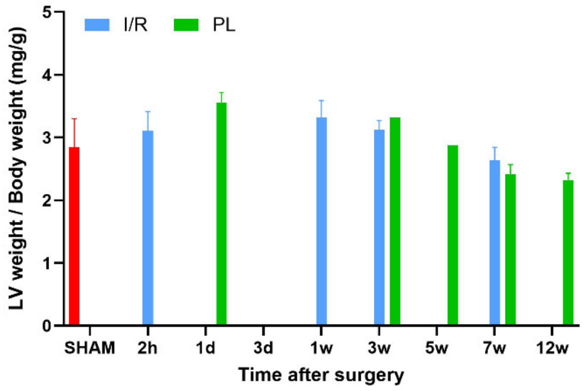

Figure S3. LV weight to body weight across groups throughout the study. Data are presented as means ± SD. Statistical significance was determined by One-Way ANOVA with Dunnett's multiple comparisons test, performed in GraphPad Prism (Version 8.1.0). h: hours, d: day, w: week.

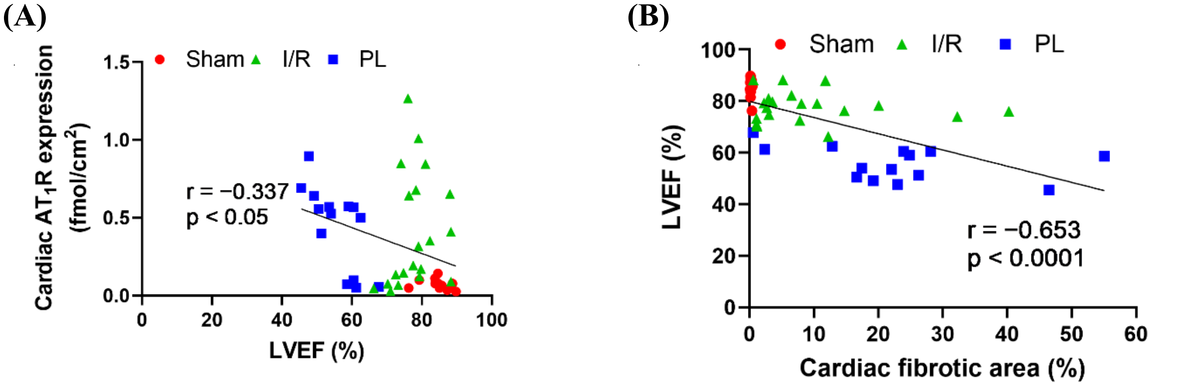

Figure S4. Correlations between (A) cardiac AT_1R expression and LVEF, and (B) LVEF and cardiac fibrotic levels in the heart of I/R and PL groups. Correlation coefficients (r) and two-tailed p-values were obtained by Pearson's correlation test using GraphPad Prism (Version 8.1.0).

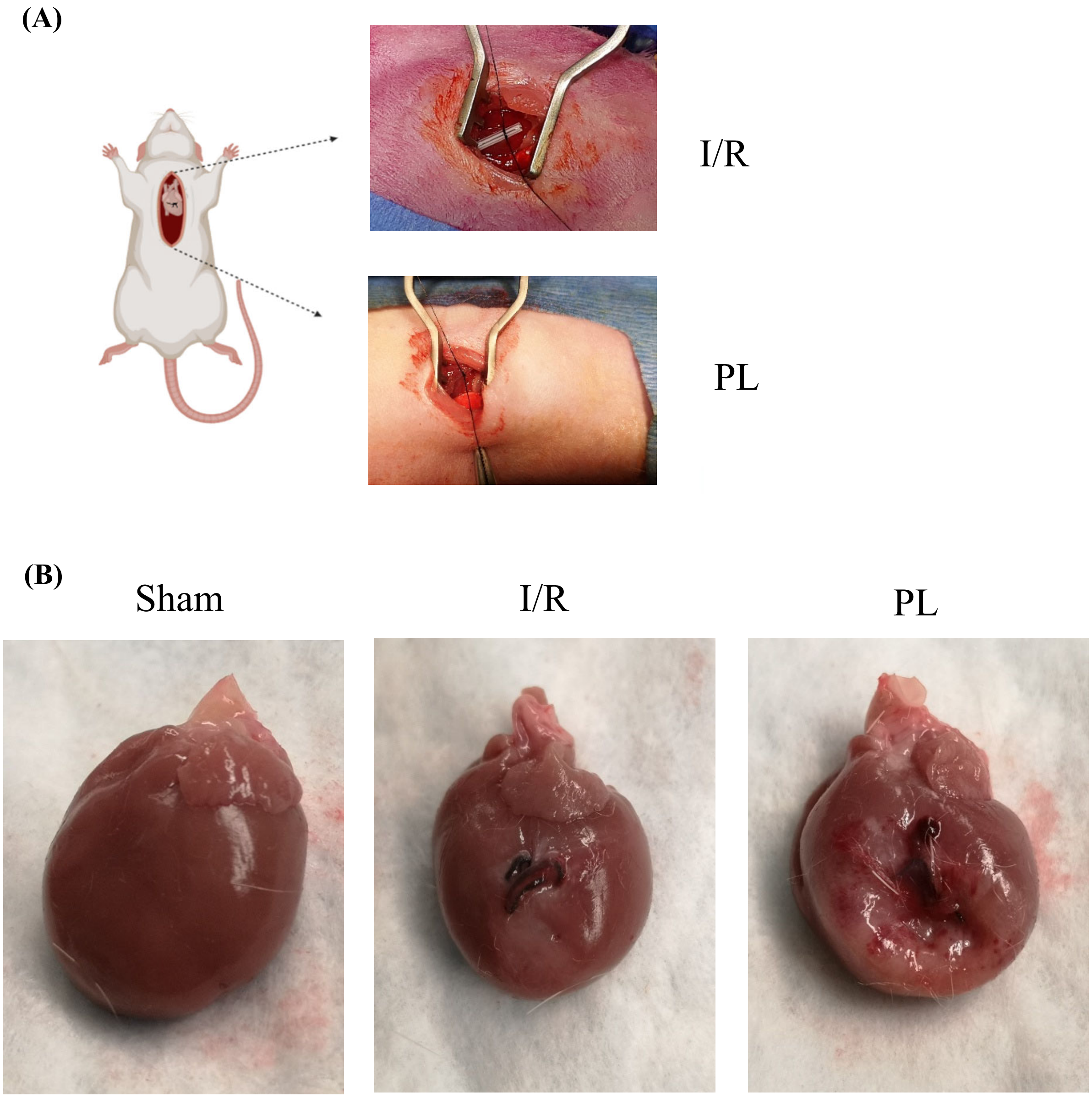

Figure S5. Animal model surgeries. (A) Representative images of I/R and PL surgeries. **(B)** Representative images of the heart at week-3 following Sham, I/R and PL surgeries.
